# Choosing real-world data for clinical and epidemiological research: methodological lessons from NHIRD and TriNetX—A narrative review

**DOI:** 10.1080/07853890.2026.2616549

**Published:** 2026-01-19

**Authors:** Teng-Li Lin, Yi-Ju Chen, Chun-Ying Wu

**Affiliations:** ^a^ Department of Dermatology, Dalin Tzu Chi Hospital, Buddhist Tzu Chi Medical Foundation, Chiayi, Taiwan; ^b^ Ph.D. Program of Interdisciplinary Medicine, National Yang Ming Chiao Tung University, Taipei, Taiwan; ^c^ Department of Dermatology, Taichung Veterans General Hospital, Taichung, Taiwan; ^d^ Department of Post-Baccalaureate Medicine, College of Medicine, National Chung Hsing University, Taichung, Taiwan; ^e^ Faculty of Medicine and Institute of Clinical Medicine, National Yang Ming Chiao Tung University, Taipei, Taiwan; ^f^ Institute of Biomedical Informatics, National Yang Ming Chiao Tung University, Taipei, Taiwan; ^g^ Health Innovation Center, National Yang Ming Chiao Tung University, Taipei, Taiwan; ^h^ Microbiota Research Center, National Yang Ming Chiao Tung University, Taipei, Taiwan; ^i^ Taiwan National Microbiota Core Facility, Taipei, Taiwan; ^j^ Division of Translational Research, Department of Medical Research, Taipei Veterans General Hospital, Taipei, Taiwan; ^k^ Department of Medical Research, Taichung Veterans General Hospital, Taichung, Taiwan; ^l^ Department of Public Health, China Medical University, Taichung, Taiwan; ^m^ National Institute of Cancer Research and Institute of Population Health Sciences, National Health Research Institutes, Maioli, Taiwan

**Keywords:** NHIRD, TriNetX, real-world evidence, observational study, big data analytics, electronic health records

## Abstract

**Introduction:**

Large-scale real-world data (RWD) are increasingly used in clinical and epidemiological research, although database-specific structures and limitations may affect study validity and applicability. The Taiwan National Health Insurance Research Database (NHIRD) and the TriNetX network are two widely used RWD sources. This review compares their key features, strengths, and limitations and discusses approaches to address methodological challenges in real-world studies.

**Discussion:**

The NHIRD comprises comprehensive, population-based, longitudinal claims data covering nearly the entire Taiwanese population. Its strengths include minimal selection bias and broad follow-up capacity. However, limitations include infrequent updates, limited clinical detail, and a Taiwan-specific context that may restrict generalizability. In contrast, TriNetX is a multinational federated network of electronic medical records from diverse healthcare systems, offering larger and more heterogeneous populations, richer clinical variables, and near real-time analytic capability, but with potential hospital-based selection bias and limited flexibility due to its fixed analytic interface. Representative studies published between 2010 and 2024 demonstrate the application of both databases across multiple medical disciplines. To mitigate data-related limitations, commonly used strategies include refined inclusion and exclusion criteria, proxy variables for unavailable measures, and triangulation with external datasets, which can strengthen study validity and interpretability.

**Conclusions:**

NHIRD and TriNetX are complementary real-world data sources, each with distinct strengths and limitations. Aligning research objectives with database characteristics is essential for appropriate study design. Recognition of platform-specific trade-offs and application of targeted methodological strategies support the validity and generalizability of real-world evidence.

## KEY MESSAGES

- The Taiwan’s National Health Insurance Research Database (NHIRD) offers comprehensive, population-based longitudinal claims with minimal selection bias but limited update frequency and Taiwan-specific scope.
- The TriNetX network is a global, federated research platform that integrates diverse populations and rich clinical information, yet it faces potential hospital-based selection bias and constrained analytic flexibility.
- Practical strategies such as refined inclusion criteria, proxy variable use, and triangulation with external sources can help researchers address these limitations and strengthen the validity of real-world studies.

## Introduction

Real-world data (RWD) refers to information routinely collected from various sources during the course of patient care, capturing patients’ actual clinical conditions [1,2]. It encompasses data from electronic health records (EHRs), insurance claims, patient registries, and even patient-reported outcomes. In medical research, RWD provides valuable insights by supporting a wide range of investigations, including disease epidemiology, variability in clinical presentations, patterns of diagnostic testing and imaging use, and risk factor analyses within specific populations [3]. Regarding disease intervention, while randomized controlled trials (RCTs) remain the gold standard for establishing efficacy and safety, they typically involve strict inclusion and exclusion criteria and relatively short follow-up periods, which limit their generalizability and the assessment of long-term outcomes [4,5]. In contrast, RWD comes from routine clinical practice and includes unselected, real-world patient populations. This helps to address the limitations of RCTs by offering insights into treatment outcomes across diverse, unselected patient populations over longer follow-up periods [6,7].

The digitization of RWD, advances in storage and computational capabilities, the maturation of standardized data models, and enhanced cross-institutional collaboration and policy support have collectively enabled large-scale data analytics [2,8]. Building on these advancements, large RWD platforms that consolidate millions of patient records now facilitate more efficient and cost-effective research than traditional clinical studies and RCTs. At the national level, several countries have built comprehensive health information repositories (HIRs) that have become critical resources for medical research. For example, Taiwan’s National Health Insurance Research Database (NHIRD) covers nearly the entire population and has been extensively used for epidemiologic studies [9]. Similarly, the Clinical Practice Research Datalink (CPRD) in the United Kingdom and South Korea’s National Health Information Database (NHID) provide rich longitudinal data that facilitate population-wide research [10,11]. On the international front, multinational RWD collaborations, such as the European Network of Centres for Pharmacoepidemiology and Pharmacovigilance and the Observational Health Data Sciences and Informatics, have long supported drug safety monitoring and enabled large-scale health data science initiatives [12,13]. More recently, emerging platforms like the TriNetX network offer federated access to electronic health records across multiple countries, providing a valuable resource for timely, large-scale observational research [14]. Collectively, these data resources help overcome limitations commonly encountered in traditional cohort studies or RCTs (Table 1), and enable a broad range of investigations into medications and health outcomes, underscoring their pivotal role in advancing clinical and public health knowledge [3].

**Table 1.** Comparison of common methodological challenges across study designs.

Methodological challenge	Large healthcare databases	Traditional cohort studies	Randomized controlled trials
Selection bias	↓ Reduced (population-based)	↑ Possible	↓ Minimized (randomization)
Non-response bias	↓ Minimal (routine data)	↑ Common	↑ Possible
Surveillance bias	↑ Possible	↑ Possible	↓ Controlled
Random error	↓ Lower (large sample size)	↑ Moderate	↑ Moderate
Loss to follow-up bias	↓ Less common	↑ Common	↑ Common
Ethical constraints	↓ Fewer issues	↑ Considerations	↑↑ Strict (informed consent)
Selection bias	↓ Reduced (population-based)	↑ Possible	↓ Minimized (randomization)

Arrows indicate relative degree: ↓reduced/lower likelihood; ↑increased/higher likelihood.

Various big data sources offer unique advantages, and the differences in their structure, types of information provided, and the scope of temporal and geographic coverage all affect their suitability for specific research goals. Several prior reviews have summarized RWD resources in Asia. For example, the Asian Pharmacoepidemiology Network provided a broad overview of multiple Asian real-world databases, with a particular focus on their application in drug-related research and pharmacoepidemiology [15,16]. In contrast, the present review adopts a different perspective by conducting a focused, side-by-side comparison of two widely used but structurally distinct data sources—Taiwan’s NHIRD and the international TriNetX network. We emphasize their respective strengths, limitations, and fit-for-purpose applications, and outline some practical strategies to address common challenges in study design and interpretation. Our discussion is not limited to drug-related research, but also encompasses study design and analytic considerations in disease epidemiology, clinical outcomes, prognosis, health economic evaluations, and risk factor analyses. By offering this overview, we aim to help researchers better understand these databases, making the most of RWD potential in generating reliable clinical evidence.

## Taiwan’s NHIRD

Taiwan’s NHIRD is a population-based claims database derived from the nation’s single-payer National Health Insurance (NHI) program, which has covered over 99% of Taiwan’s 23 million residents since its inception in 1995 (Table 2) [9]. Originally designed for administrative and reimbursement purposes, the NHIRD has become a widely used HIR for epidemiologic and health services research [17–20]. Its national coverage across all levels of healthcare enhances the representativeness and generalizability of findings.

**Table 2.** Comparison of NHIRD and TriNetX in database structure and characteristics.

Feature	NHIRD	TriNetX
System openness	Closed, nationwide (Taiwan only)	Open, international network
Category	Health insurance repository	Clinical data repository
Source	National health insurance claims	Electronic health records
Population size	∼23 million	∼200 million (as of December 2025)
Data range	From 1997 onward	Mostly from 2000 onward (varies by HCOs)
Data Renewal	Every year	Every 1–4 weeks
Data linkage	Can be linked to national registries	Limited linkage (varies by HCOs & region)
Data contents		
Demographics	Available	Available (including race & ethnicity)
Vital signs	Unavailable	Available
Diagnoses	Available (with physician specialty)	Available
Procedures	Available	Available
Medications	Available (including dosage & duration)	Available
Lab results	Available (from the integrated NHILD)	Available
Genomics	Available (linking to Taiwan Biobank)	Available (in some sites)
Household	Available	Unavailable

NHIRD: the National Health Insurance Research Database; HCOs: health care organizations.

To facilitate research while safeguarding privacy, the NHIRD data are de-identified and encrypted before release. All identifiable personal information—including names, identification numbers, and precise dates of birth—is removed using standardized anonymization protocols [9,21]. The database primarily consists of structured administrative claims data and does not include narrative clinical documentation. Original service dates are preserved to support longitudinal and time-specific analyses. Researchers can only access the NHIRD through on-site terminals at Taiwan’s Health and Welfare Data Science Center (HWDC), a government-run, secure computing environment that strictly controls data access and prohibits data extraction or transfer.

The NHIRD comprises the full population-level database. In addition, it includes longitudinal subsets known as the Longitudinal Health Insurance Databases (LHIDs), which comprise claims data from one million individuals randomly sampled from the full NHIRD population. These subsets retain demographic and healthcare utilization characteristics that closely resemble to the entire database [9,21]. While earlier studies frequently relied on LHIDs for research convenience, access to the full NHIRD population is available for approved research projects through the HWDC. Since 2016, NHIRD data can also be linked through the HWDC to other biomedical datasets, such as National Health Insurance Laboratory Database (NHILD), cancer registries, mortality files, and rare disease databases. These linkages allow for multi-dimensional longitudinal analyses across a range of research areas. The accuracy of diagnostic and procedural coding in the NHIRD has been validated in multiple studies [22,23], further supporting its reliability for clinical research.

The NHIRD provides a wide array of structured data elements essential for real-world evidence research. Available variables include patient demographics (e.g. age, sex, place of residence), diagnostic codes based on the International Classification of Diseases, Ninth Revision (ICD-9-CM) before 2016 and the Tenth Revision, Clinical Modification (ICD-10-CM) thereafter, prescription and dispensation records, surgical and procedural codes, healthcare utilization, and detailed medical expenditure data (Table 2) [21]. Information on laboratory and diagnostic test orders—such as test types and dates—is also recorded, and corresponding test results may be available through linkage with the NHILD. Other clinical data, such as body weight, height, imaging findings, family history, and lifestyle factors (e.g. smoking, alcohol use), are not captured.

Similar to Taiwan’s NHIRD, other Asian national claims databases include South Korea’s NHID and Japan’s National Database of Health Insurance Claims and Specific Health Checkups (NDB) [11,24]. These databases are also derived from single-payer health insurance systems, capture structured administrative claims data for nearly the entire population, and support longitudinal, population-based research. Consequently, they are often considered alternative sources to NHIRD for similar study designs. Nevertheless, each database has its distinctive features: for example, NHIRD allows more comprehensive cross-linkage with other registries, whereas NHID and NDB may offer larger sample sizes [25]. Overall, given the completeness of its structure and the extensive use in research over many years, NHIRD is one of the most well-established and classic examples of a large national claims database.

## TriNetX network

TriNetX is a global collaborative health research platform that, as of December 2025, includes de-identified, real-time EHR data from more than 200 million patients across 170 healthcare organizations (HCOs) in over 20 countries [14]. Participating HCOs contribute de-identified patient data in exchange for access to the platform’s large-scale analytics tools and opportunities to participate in industry-sponsored studies. In turn, industry sponsors support the development of the platform in exchange for access to network data that can improve the efficiency of clinical trial planning and execution. This model has allowed TriNetX to overcome the common funding challenges associated with building and maintaining clinical data repositories (CDRs).

The TriNetX Network comprises multiple regional and specialty sub-networks, primarily drawing data from the United States, with additional contributions from South America, Europe, the Middle East, Africa, and the Asia-Pacific region. Its main sub-networks include the US Network, the EMEA (Europe, Middle East, and Africa) Network, the APAC (Asia-Pacific) Network, the LATAM (Latin America) Network, and the Global Network, which integrates data from all participating HCOs worldwide [14]. To maintain confidentiality, the identities of contributing HCOs and their data sources are not disclosed.

TriNetX adheres strictly to data privacy regulations such as the Health Insurance Portability and Accountability Act (HIPAA), the General Data Protection Regulation, and the Lei Geral de Proteção de Dados. Patient data are handled in a HIPAA-compliant manner, with additional safeguards applied as required by local data protection regulations; patient identifiers are replaced by synthetic identifiers used solely for research purposes, ages over 90 years are not disclosed, and ZIP codes are excluded. Calendar dates of clinical events are obfuscated through date shifting, whereby dates may be shifted forward or backward by a fixed interval (ranging from 0 to 365 days) [26], while preserving the relative temporal relationships among events for an individual patient. In addition, TriNetX has created smaller networks in which dates are not shifted or are minimally shifted (±7 days), which may be more suitable for studies requiring alignment with specific calendar time points, such as investigations of the COVID-19 pandemic or seasonal epidemics. Only aggregated data are shared for research purposes, and studies using the platform are often exempt from institutional review board oversight.

TriNetX integrates a wide range of standardized data types, including demographics, diagnoses, procedures, medications, laboratory results, and genomics (Table 2), using internationally recognized coding systems such as ICD-10 (and ICD-9 for older records) and Logical Observation Identifiers Names and Codes [14,27]. In addition to structured EHR elements, TriNetX also leverages natural language processing (NLP) techniques to extract information from unstructured clinical narratives, such as physician notes and radiology reports [28]. Large language models (LLM) further enhance these NLP capabilities by enabling more context-aware and scalable information extraction from free-text data. The extracted information is often precomputed and transformed into structured data elements, allowing for more efficient, reproducible querying and downstream analyses. These structured EHR data can also be linked with external data sources, including insurance claims and mortality records. The platform then provides a unified interface that harmonizes heterogeneous data across institutions and supports various research capabilities, including cohort comparisons, outcome analyses, treatment pathway exploration, and disease incidence estimation [27].

By 2024, TriNetX had enabled nearly 20,000 sponsored clinical trial opportunities and contributed to over 1,000 peer-reviewed publications, including articles in high-impact medical journals [29–31]. During the COVID-19 pandemic, TriNetX played a pivotal role in providing RWD to rapidly address critical clinical and epidemiological questions [32–34], thereby supporting timely adaptations in clinical practices and public health policies.

## Comparative strengths of NHIRD and TriNetX

Both NHIRD and TriNetX share several key advantages inherent to big data research (Table 3). First, studies using these databases are highly cost-effective. Since the data have already been collected and curated, research that might otherwise take years to complete can be conducted more efficiently, enabling the investigation of rare exposures or outcomes [35,36], as well as insights into conditions that require long-term observation [37,38]. Second, the vast volume of data provides strong statistical power [39,40]. When combined with matching and adjustment methods, it helps mitigate confounding bias and enhances the reliability of research outcomes. Third, researchers can perform detailed subgroup analyses, including populations often excluded from clinical trials—such as the young children [41], elderly [42], or pregnant individuals [43]—thereby supporting the development of personalized medicine strategies. These retrospective analyses are also particularly valuable in addressing clinical questions that would be unethical or infeasible to examine through prospective trials [44,45]. Fourth, because these platforms are based on RWD, they better reflect the complexity of actual clinical practice, increasing the generalizability of findings [33,39,46]. For intervention-related studies, they offer a more comprehensive perspective on treatment effectiveness and safety in routine care settings [17,18,20,31,37,38,42,44], filling important gaps left by RCTs.

**Table 3.** Shared and unique strengths and limitations of NHIRD and TriNetX.

Category	Common Features	NHIRD	TriNetX
Strengths	- Cost-effective and efficient - Large datasets with strong power - Suitable for rare diseases/exposures - Long-term follow-up - Real-world setting	- Nationwide coverage - Standardized coding - Detailed data (e.g. diagnosis dates, doses, specialties) - Linkable to other databases - Enable flexible study designs	- Large, diverse, global cohort - Broad data types (labs, vitals, genomics) - Rapid updates - Analyses without programming
Limitations	- Observational design (no causality) - Coding errors, missing data - Lacks PROs, lifestyle, adherence - Residual confounding - Big data paradox	- Taiwan-only - Missing vital signs - Relatively slow updates - Requires programming	- Hospital-based selection bias - Inconsistent coding practices - Less granular clinical data - Cannot link to other databases - Fixed interface with limited design flexibility
Example Suitable Research Questions		- Disease incidence, prevalence, and mortality studies - Detailed drug utilization analyses (dose, duration, adherence) - Cross-generational analyses or linkage with other Taiwanese databases	- Multi-national or multi-ethnic comparative studies - Extremely rare events - Evaluation of newly introduced therapies or technologies

NHIRD: National Health Insurance Research Database; PROs: patient-reported outcomes.

While similar in many respects, the NHIRD offers several distinct strengths over TriNetX (Table 3). Derived from Taiwan’s NHI system, the NHIRD captures virtually all outpatient visits, hospitalizations, prescriptions, and procedures nationwide [9,21]. This near-universal coverage within a closed-loop system minimizes selection bias and ensures consistent, standardized coding practices across the healthcare continuum. Its centralized architecture also allows seamless linkage with other government-held databases, supporting rich and long-term longitudinal analyses [47–49]. Moreover, the NHIRD provides more granular clinical and administrative details, such as exact diagnosis dates [23], physician specialties [50,51], precise dosing and duration of prescriptions [20,41], and even household-level information [52]—features typically unavailable in TriNetX. For studies requiring laboratory data, longitudinal test results can also be accessed through the integrated NHILD, providing critical support for research [53]. Combined with its superior analytic flexibility, which allows researchers to write and execute customized code directly on raw data extracts, the NHIRD supports a wide range of study designs beyond cohort studies, including prevalence and incidence estimation [48], cross-sectional or case-control studies [23,50], drug utilization research [44], cost – effectiveness analyses [54], and even predictive modeling [39,46].

In contrast, TriNetX offers several advantages over the NHIRD (Table 3). As a global platform aggregating EHR data from numerous HCOs, TriNetX provides access to a larger and more diverse sample. Combined with NLP-enabled extraction of information from textual sources, this expanded data depth offers sufficient statistical power even for studies of extremely rare exposures or outcomes [28,36,55]. Its multinational coverage and availability of race and ethnicity data enable comparative analyses across different regions and populations [38,56]. With updates occurring every 1 to 4 weeks, the platform is well suited for monitoring emerging diseases [32–34], new treatments [38,57], or changes in clinical practice in near real time. The inclusion of vital signs [58], laboratory values [38,57], and genomic data [59] also helps address a common limitation seen in HIRs and CDRs, where such clinical parameters are often unavailable. Moreover, TriNetX features a user-friendly analytical pipeline with several predefined methods that enable researchers to perform sophisticated analyses—such as cohort selection, propensity score matching, and Kaplan–Meier survival analysis—without requiring advanced programming or data science skills [14,27,28]. This intuitive interface broadens accessibility to clinical investigators and facilitates efficient real-world evidence generation.

## Limitations of NHIRD vs TriNetX

Despite their considerable strengths, NHIRD and TriNetX also share several common limitations seen in large-scale databases (Table 3). First, these sources provide observational data, so analyses can identify associations but cannot establish causality. Second, since these data come from routine clinical care, issues such as coding errors, misclassification, variability in clinical practice, and missing data elements may affect data accuracy. This reflects what van der Lei referred to as the ‘First Law of Informatics,’ which cautions the limitations of using routinely collected data for research purposes (see Panel 1 for definition) [60]. Third, as these data were not originally gathered for research, certain important variables are often unavailable, including patient-reported outcomes, lifestyle factors, and social determinants of health. Prescription records reflect only what was prescribed, not what was actually taken, making it difficult to determine patients’ medication adherence from the database. Furthermore, patients in real-world setting may experience intercurrent events (ICEs) (see Panel 1 for definition), including treatment-modifying events (e.g. treatment discontinuation, switching, or use of rescue therapies) and truncating events (e.g. death), which can affect either the interpretation or the existence of the outcome of interest [61,62]. Fourth, due to the lack of randomization, findings may be skewed because of unmeasured confounders. Although various statistical methods are often employed to balance group differences, such adjustments also lead to sample attrition. Finally, while large datasets can enhance statistical power, they sometimes may may amplify spurious associations—a phenomenon referred to as the ‘big data paradox’ (see Panel 1 for definition) [63]. Researchers should interpret results cautiously and place greater emphasis on results that are not only statistically significant but also clinically meaningful.

NHIRD has certain limitations that set it apart from TriNetX (Table 3). Since its data are derived exclusively from Taiwan’s NHI system, the database reflects the structure, reimbursement policies, and clinical practice patterns of the Taiwanese healthcare system and primarily represents the Taiwanese population. As a result, findings generated from NHIRD are most applicable to Taiwan and may have limited generalizability to other healthcare systems or multi-ethnic populations. Access to NHIRD is also limited to researchers based in Taiwan or their collaborators, creating an entry barrier. In addition, because NHIRD is an administrative claims database, diagnostic coding may be influenced by reimbursement policies, potentially resulting in ‘upcoding’ that overstates disease severity for billing purpose [9]. Although the database is typically updated on an annual basis, there may be a substantial time lag between healthcare delivery and data availability for research use, which makes NHIRD less suitable for studying emerging diseases or newly approved treatments. Moreover, its coverage is also confined to services reimbursed by the NHI system, excluding out-of-pocket medications or therapies, which may limit its usefulness in certain research domains [21]. Finally, although the NHIRD contains rich data, users must write their own analytical code to extract and analyze information. This task requires advanced programming skills and may present a barrier for some researchers.

Compared with NHIRD, TriNetX also has its own limitations (Table 3). As a multinational federated network, TriNetX reflects heterogeneous healthcare systems, clinical practice patterns, and coding conventions across participating countries. Such between-country variation may influence data consistency and comparability, particularly when stratified analyses by country or data partner are not feasible. Because it relies solely on data from participating HCOs rather than a predefined population, the representativeness of the database is not fully known, introducing an inherent risk of selection bias [64]. Its open-network nature also means that any care patients receive outside of these HCOs goes unrecorded. This may result in incomplete follow-up with left-, right-, or interval-censoring when patients seek treatment across multiple systems (see Panel 1 for definitions) [65,66]. Additionally, variations in data protection regulations in different countries may cause inconsistencies in coding practices [67]. And although TriNetX aggregates data globally, over 60% comes from the United States. Data detail and quality also vary by region, with richer granularity from U.S. HCOs and more limited data in other countries. NLP and LLM-based extraction of unstructured clinical notes, despite enhancing data depth, is subject to spelling errors, variability in documentation, and heterogeneity across clinicians [28]. Furthermore, while it contains diverse data types, some key details are sparse, such as diagnosis dates, physician specialties, total medication doses and durations, longitudinal laboratory values, and household-level information. Researchers also can’t link TriNetX records to other datasets, which narrows the scope for broader data integration. Outside the U.S., privacy rules generally prevent downloading raw datasets requiring analyses to be conducted through TriNetX’s built-in fixed interface. For U.S.-based datasets, however, patient-level data may be exported under specific conditions, including submission of an IRB-approved protocol and approval through TriNetX’s data access request process; however, such access remains restricted and is not universally available [27].

## Strategies to address limitations in NHIRD and TriNetX

When analyzing large-scale databases like NHIRD and TriNetX, several approaches can help address their inherent limitations (Table 4). Although observational studies cannot establish causality, applying Hill’s criteria [68]—such as examining the consistency of associations across different populations [69] and demonstrating biological gradients [57]—can strengthen causal inference (see Panel 1 for summary). For pharmacoepidemiological research, advanced methods like target trial emulation replicate key features of RCTs, thereby improving the validity of causal estimates (see Panel 1 for definition) [70,71]. To reduce coding errors and misclassification, researchers can improve data accuracy by refining case definitions, such as requiring multiple coded diagnoses [50] or excluding patients with conditions that could mimic the target diagnosis [69]. Missing variables can sometimes be approximated using proxy measures; for instance, prescriptions for later-line medications may indicate more severe disease [57], and codes for nicotine dependence (ICD-10-CM: F17), alcoholic liver disease (K70), or psychosocial hardships (Z55–Z65) can partially reflect smoking and drinking behaviors or socioeconomic status [38,40]. Since medication compliance is typically unknown in these databases, drug treatment studies should be interpreted as intention-to-treat analyses (see Panel 1 for definition) [38,56]. To address ICEs, the ICH E9(R1) estimand framework emphasizes the need to explicitly define the estimand (see Panel 1 for definition), thereby clearly specifying the research question [61,62]. Based on the defined estimand, analytic strategies for anticipated ICEs may be prespecified, including treatment policy, composite, while-on-treatment, hypothetical, or principal stratum approaches (see Panel 1 for definition) [61,62]. Limiting study populations, applying matching techniques, and conducting subgroup analyses can further help reduce confounding. Finally, to avoid the ‘big data paradox,’ analyses should be based on prior experimental evidence, biological plausibility, or clinical rationale rather than purely data-driven fishing expeditions.

**Table 4.** Strategies to address limitations in the NHIRD and TriNetX.

Limitation	Potential solutions
Common limitations	
Inability to establish causality	- Design studies using Hill’s criteria or target trial emulation
Coding errors; misclassification	- Apply strict case definitions - Exclude diagnoses requiring differential diagnosis
Missing variables	- Use proxy measures (e.g. treatment response as a surrogate for disease severity)
Unknown medication adherence	- Interpret analyses as intention-to-treat
Confounding	- Restrict populations - Apply matching techniques - Conduct subgroup analyses
Big data paradox	- Base research on prior experimental evidence, biological plausibility, or clinical rationale
NHIRD-specific	
Restricted to Taiwanese population	- Validate findings with other databases outside Taiwan
For unavailable lab results	- Use repeated tests as proxies for abnormality - Link with other local databases
TriNetX-specific	
Potential selection bias	- Focus on higher-quality subsets (e.g. TriNetX US Network) - Conduct subgroup analyses
Unknown representativeness	- Examine cohort-level summary statistics (e.g. geographic distribution) - Conduct stratified analyses
Missing data in open networks	- Clearly define observation windows
Fixed analytical interface	- Perform multiple sensitivity analyses under different assumptions

NHIRD: National Health Insurance Research Database.

For NHIRD, although its research data are restricted to the Taiwanese population, findings can still be strengthened by validating results against other regional or international datasets to enhance generalizability (Table 4) [46]. This comparative approach allows NHIRD to remain highly valuable despite its geographic scope. Potential issues related to ‘upcoding’ can be mitigated by applying stricter case definitions—for example, requiring diagnoses to be accompanied by supporting tests or relevant treatments [72], or restricting cases to those also listed in more rigorous NHIRD’s sub-databases such as the Registry for Catastrophic Illness Patients [17]. Where detailed laboratory results are unavailable, researchers can use real-world clinical behaviors as proxy indicators. Frequent repetition of a particular laboratory test, for instance, may suggest abnormal findings [72], while long-term anti-inflammatory prescriptions can serve as a marker for chronic musculoskeletal inflammation in patients with rheumatic diseases [73]. Additionally, NHIRD can be linked with other national databases in Taiwan—such as the NHILD, Taiwan Biobank, Taiwan Cancer Registry, National Student Fitness Tests Database, and Air Quality Monitoring System Database—to supplement unavailable data [47,49,53]. These linkages help fill data gaps and expand the research possibilities using NHIRD.

For TriNetX, researchers can reduce potential selection bias by focusing on subsets of HCOs with more uniform data quality and completeness (Table 4). Cohort-level summary statistics—such as patient geographic distribution—can be used to contextualize the coverage of contributing HCOs within a given cohort and to better understand potential limitations in representativeness. Subgroup analyses—such as stratification by geographic region or patient characteristics—or cross-validation with other databases can further address heterogeneity and improve the interpretability of results [38,74]. Clearly defining observation windows—such as specifying baseline periods before the index date and setting appropriate follow-up intervals—may help mitigate bias arising from missing data in open-network systems [57]. When data lack fine-grained detail, the available information can still yield meaningful preliminary insights. For example, even though time-varying exposures cannot be assessed, time-fixed medication analyses can still provide early evidence on treatment–outcome relationships [38,57]. Similarly, while only the most recent laboratory value before the index date is captured, it still reflects the patient’s baseline status at cohort entry [69]. Finally, although the platform’s fixed analytical interface restricts flexibility in study design, researchers can compensate by conducting multiple sensitivity analyses under different assumptions [56]. The built-in tools facilitate efficient, repeatable testing of alternative designs to explore the robustness of findings.

## Fit-for-purpose comparison between NHIRD and TriNetX using an example research question

Due to their respective strengths and limitations, NHIRD and TriNetX are each better suited for specific types of research. Some illustrative examples are provided in Table 3. The Structured Process to Identify Fit-for-Purpose Data (SPIFD) tool can help guide the selection [75]. Here, we use a recent clinical question as a hypothetical example: whether biologics with different mechanisms of action differ in their risk of incident psoriatic arthritis (PsA) among patients with psoriasis after treatment, and we apply the SPIFD framework to highlight the differences in suitability of these two databases across key dimensions.

Following the SPIFD framework, Step 1 involves operationalizing the minimal criteria required to answer the research question into concrete, assessable elements and ranking them according to their importance for study validity. Common elements include eligibility criteria, exclusion criteria, treatment definition, outcome definition, key potential confounders, and minimum follow-up duration. Among these, the criteria ranked as most important (highest-ranking criteria) for this research question are the ability to precisely define treatment initiation for biologics with different mechanisms, and to accurately identify psoriasis and PsA to correctly define the study population, exclude patients with baseline PsA, and ascertain incident PsA outcomes.

In Step 2, researchers use the highest-ranking criteria from Step 1 to identify databases suitable for the study. Typically, this step narrows the list to 3–5 candidate sources. In this example, both NHIRD and TriNetX meet the highest-ranking criteria and are therefore included for further assessment.

In Step 3, a detailed feasibility assessment is conducted for each candidate data source. While both databases meet the essential design requirements, TriNetX offers specific advantages for this research question. Its data include patients who have received biologics beyond those covered by specific reimbursement criteria, are updated more frequently—allowing the inclusion of newer biologic agents—and provide faster access and shorter analysis timelines. Therefore, for this particular research question, TriNetX may be the more suitable data source (Table 5) [25,76,77].

**Table 5.** Example of assessing a hypothetical research question (risk of psoriatic arthritis in psoriasis patients treated with biologics of different mechanisms) using the SPIFD framework: Comparison between NHIRD and TriNetX (step 3 detailed data feasibility assessment).

Design element	Requested information	NHIRD	TriNetX
Study population	Criteria for identifying psoriasis patients; exclusion of baseline PsA; demographic characteristics; inclusion/exclusion codes or algorithms	Yes	Yes
Treatment/exposure group and comparator group(s)	Start date and type of biologic therapy; treatment codes; completeness of exposure capture	Yes, but incomplete as only NHI-reimbursed treatments captured	Yes
Primary outcomes	Incident PsA identification (diagnosis codes); completeness of outcome capture; timing relative to treatment initiation	Yes	Yes, specialty of diagnosing physician unknown
Length of follow-up and data recency	Minimum follow-up time per patient; data cutoff date; frequency of database updates	Yes, updated annually	Yes, updated every 1–4 weeks
Confounding variables	Key potential confounders (e.g. age, sex, comorbidities, prior treatments); availability and completeness of covariate data; coding algorithms or definitions	Yes	Yes
Data access considerations	Time to data access; time to analyze	Moderate	Fast
Final data source selection			✓

SPIFD: Structured Process to Identify Fit-for-Purpose Data; NHIRD: National Health Insurance Research Database; NHI: National Health Insurance; PsA: psoriatic arthritis.

## Future opportunities and outlook

Looking ahead, researchers may benefit from combining the distinct advantages of national databases like NHIRD with those of international platforms such as TriNetX. By combining NHIRD’s population-level coverage with TriNetX’s larger samples and broader range of data elements, the limitations of each individual data source can be overcome. This kind of integrative approach allows for cross-validation of findings and enables the use of hybrid modeling techniques to explore differences between cohorts more thoroughly. Such integrated data efforts could generate more generalizable results, which can then inform treatment guidelines across diverse populations, and also enable more detailed subpopulation analyses to support personalized care in routine clinical settings.

Beyond observational analysis, the harmonization of NHIRD and TriNetX data can be used to train predictive algorithms—both at the patient and population level. For example, generative models could help estimate the risk of disease progression, supporting earlier interventions. Longitudinal claims data combined with clinical details may help simulate disease trajectories and contribute to the development of digital health tools. Lastly, multinational and population-based analyses can facilitate more nuanced evaluations of cost-effectiveness across different healthcare systems. Such insights may help shape health policies that are better suited to local healthcare systems and needs.

## Conclusion

In conclusion, both NHIRD and TriNetX offer highly valuable real-world data, each with its own advantages and limitations. Understanding these differences is crucial for designing rigorous analyses and appropriately interpreting findings derived from these sources. By applying suitable research methods tailored to the characteristics of each database, adopting effective strategies to address their limitations, and even integrating both datasets, researchers can conduct more robust studies and generate meaningful evidence to support clinical practice and inform health policy. As data quality continues to improve and analytical techniques become increasingly advanced, the full potential of real-world data can be more effectively realized. This progress will not only enhance future research but ultimately help improve patient care.

## Figures and Tables

**Panel 1.** Conceptual summary of terms referenced in this article.

Term	Explanation
van der Lei’s First Law of Informatics	States that ‘data should not be used for purposes other than those for which they were collected.’ This law emphasizes the potential limitations and biases when using routine clinical data for secondary research purposes.
Intercurrent Events	Events that occur after baseline and can influence either the interpretation or the availability of outcome data. These events are generally classified into two main types: those that modify treatment and those that truncate the observation, such as death.
Big Data Paradox	Refers to the phenomenon where large sample sizes improve statistical power but may also amplify bias or spurious associations, leading to misleading conclusions if data quality is poor or unrepresentative.
Left-, Right-, and Interval-Censoring	Types of incomplete time-to-event data. - *Left-censoring*: Event occurred before observation began. - *Right-censoring*: Event not yet occurred at end of follow-up. - *Interval-censoring*: Event occurred between two known time points, but exact time is unknown.
Hill’s Criteria for Causality	A framework of nine principles proposed by Sir Austin Bradford Hill to guide the assessment of causality in observational studies. While not all must be met, the more that are fulfilled, the stronger the case for a causal relationship. These include: *Temporality, Strength, Consistency, Specificity, Biological Gradient, Plausibility, Coherence, Experiment, Analogy.*
Target Trial Emulation	A methodological approach to designing observational analyses that mimic the structure of a hypothetical randomized trial by specifying eligibility criteria, treatment assignment, follow-up period, and outcome definitions.
Intention-to-Treat Analysis	An analysis strategy in randomized or emulated trials where participants are analyzed based on their original treatment assignment, regardless of adherence. Such approach maintains randomization benefits and avoids treatment-related bias.
Estimand	A clearly specified definition of the treatment effect that the study seeks to estimate.
Strategies for handling intercurrent events	Five strategies for intercurrent events - *Treatment policy*: Outcomes are analyzed regardless of whether the intercurrent event occurs. - *Composite*: The intercurrent event is incorporated into the outcome definition. - *While-on-treatment*: Outcomes are evaluated up to the occurrence of the intercurrent event. - *Hypothetical*: Outcomes are defined under a scenario in which the intercurrent event does not occur (or does occur). - *Principal stratum*: Outcomes are assessed within the subgroup of patients who would (or would not) experience the intercurrent event.

## Data Availability

Data sharing is not applicable to this article as no data were created or analyzed in this study.
